# Inter-Institutional Comparison of Personalized Risk Assessments for Second Malignant Neoplasms for a 13-Year-Old Girl Receiving Proton *versus* Photon Craniospinal Irradiation

**DOI:** 10.3390/cancers7010407

**Published:** 2015-03-10

**Authors:** Phillip J. Taddei, Nabil Khater, Rui Zhang, Fady B. Geara, Anita Mahajan, Wassim Jalbout, Angélica Pérez-Andújar, Bassem Youssef, Wayne D. Newhauser

**Affiliations:** 1 Division of Radiation Oncology, The University of Texas MD Anderson Cancer Center, Unit 1202, 1515 Holcombe Blvd, Houston, TX 77030, USA; E-Mails: amahajan@mdanderson.org (A.M.); perezandujara@radonc.ucsf.edu (A.P.-A.); 2 The University of Texas Graduate School of Biomedical Sciences at Houston, P.O. Box 20334, Houston, TX 77225, USA; 3 Department of Radiation Oncology, American University of Beirut Medical Center, P.O. Box 11-0236, Riad El Solh, Beirut 1107 2020, Lebanon; E-Mails: fg00@aub.edu.lb (F.B.G.); wj01@aub.edu.lb (W.J.); by04@aub.edu.lb (B.Y.); 4 Department of Radiation Oncology, Hôtel-Dieu de France Hospital, University of St. Joseph, P.O. Box 166830, Alfred Naccache Blvd, Beirut, Lebanon; E-Mail: nabil.khater@hdf.usj.edu.lb; 5 Medical Physics Program, Department of Physics and Astronomy, Louisiana State University, 202 Nicholson Hall, Tower Dr., Baton Rouge, LA 70803, USA; E-Mails: rzhang@marybird.com (R.Z.); newhauser@lsu.edu (W.D.N.); 6 Department of Physics, Mary Bird Perkins Cancer Center, 4950 Essen Lane, Baton Rouge, LA 70809, USA

**Keywords:** pediatric medulloblastoma, second malignant neoplasm risk, craniospinal irradiation, health care disparities, proton therapy

## Abstract

Children receiving radiotherapy face the probability of a subsequent malignant neoplasm (SMN). In some cases, the predicted SMN risk can be reduced by proton therapy. The purpose of this study was to apply the most comprehensive dose assessment methods to estimate the reduction in SMN risk after proton therapy *vs.* photon therapy for a 13-year-old girl requiring craniospinal irradiation (CSI). We reconstructed the equivalent dose throughout the patient’s body from therapeutic and stray radiation and applied SMN incidence and mortality risk models for each modality. Excluding skin cancer, the risk of incidence after proton CSI was a third of that of photon CSI. The predicted absolute SMN risks were high. For photon CSI, the SMN incidence rates greater than 10% were for thyroid, non-melanoma skin, lung, colon, stomach, and other solid cancers, and for proton CSI they were non-melanoma skin, lung, and other solid cancers. In each setting, lung cancer accounted for half the risk of mortality. In conclusion, the predicted SMN risk for a 13-year-old girl undergoing proton CSI was reduced *vs.* photon CSI. This study demonstrates the feasibility of inter-institutional whole-body dose and risk assessments and also serves as a model for including risk estimation in personalized cancer care.

## 1. Introduction

In 2012, approximately 163,000 children aged 14 and younger were diagnosed with cancer worldwide [1], about half of whom received radiotherapy. Eighty percent of these children lived in developing countries [2] that do not offer advanced radiotherapy techniques, such as proton therapy, that are capable of reducing the dose in non-tumor tissues *vs.* traditional radiation therapies [3,4]. The 5-year survival rate for children with cancer is high—83% in the U.S. [5] and lower but improving in developing countries [2]. However, whether treated in developed or developing countries, childhood cancer survivors face the likelihood of long-term effects from their treatment that can be debilitating, chronic, and even fatal. Children receiving radiotherapy are particularly vulnerable compared to adults because they have a higher sensitivity to many of these radiation effects, their organs are closer to the treatment fields, and they have longer expected survival times. The late effect of greatest concern for children who receive radiotherapy is the development of a treatment-related subsequent malignant neoplasm (SMN) [6]. The use of proton radiotherapy for treating children with curable solid tumors to reduce the risk of late effects such as SMN is expanding rapidly in North America, Europe, and Asia [7] but is not currently available in South America, Africa, and Australia, including the regions of Central America, Southeast Asia, and the Middle East [8].

By applying risk models and accurate dosimetry, the lifetime risks of SMN can be estimated for individuals and populations that were exposed to radiation. Some studies have estimated the risk of SMN for children receiving radiotherapy for tumors of the central nervous system [9,10,11,12,13,14], the second most common site of childhood cancer worldwide. These studies estimated the radiation dose in organs and tissues from therapeutic radiation within the treatment fields and from leakage and scatter radiation outside the fields. Some studies have compared proton *vs.* photon therapy within a single institution in terms of the predicted risk of secondary effects [11,12,14,15,16,17,18,19], each of which found proton therapy to be superior to photon therapy, with fewer radiotherapy-related toxicities and equal tumor control [20]. Over the past 12 years, the methods of evaluating the radiation dose throughout the patients’ bodies have evolved, and now a comprehensive estimate of SMN risk based on these advanced dosimetric methods is achievable, and this can be done inter-institutionally. Until now, a comparison in SMN risks had not been made between two institutions, one having proton therapy and the other having only photon therapy, for a child receiving radiotherapy for brain cancer. Such studies are needed to generate evidence to inform treatment decisions and planning in regions in which proton therapy is not locally available. For these reasons, using the most advanced and comprehensive dose reconstruction methods to estimate SMN risks, we compared a best-available radiotherapy in an academic cancer center in the Middle East (photon therapy) with that available in an academic cancer center in the U.S. (proton therapy) to treat a pediatric brain cancer.

The purpose of this study was to estimate for a single patient the reduction in SMN risk that may be achieved by applying proton beams rather than photon beams in craniospinal irradiation (CSI). We did this by estimating the predicted risks of radiogenic SMN incidence and mortality for a 13-year-old girl in two separate clinical environments. These estimates included dosimetric contributions from both therapeutic and stray radiation calculated in this study and widely-accepted SMN risk models from the literature. As a result, we were able to determine the SMN sites of greatest concern for each modality. In addition, in this multi-institutional study, we expanded, tested, and improved our methods for estimating out-of-field dose in photon radiotherapy, which will be applied in future studies.

## 2. Methods and Materials

### 2.1. Patient and Treatment Objectives

To test a typical scenario for a pediatric cancer patient with prospects for long-term survival of a primary cancer, we created separate CSI treatments for a patient diagnosed with medulloblastoma according to the standards of care of two leading academic cancer treatment facilities in their respective regions—The University of Texas MD Anderson Cancer Center (“MD Anderson”) using protons [21] and the American University of Beirut Medical Center (AUBMC) using photons. Unlike MD Anderson, AUBMC offers only photon therapy and not proton therapy, a limitation that is true of cancer care facilities throughout the Middle East and all developing countries (China and South Africa are the only countries classified as developing by the World Bank in which patients have been treated with proton therapy, but they have accumulated less than 2,000 patients treated to date) [8,22]. The intent of the CSI was to sterilize potential residual cancer cells in the tumor bed, central nervous system, and cerebrospinal fluid by delivering the prescribed dose, *D_Rx_*, of 23.4 Gy-RBE to the entire craniospinal axis (1.8 Gy-RBE/fraction in 13 fractions). The patient was a 13-year-old girl, with a height of 147 cm and a mass of 42.6 kg, who had been treated with proton CSI at MD Anderson. As part of the patient’s care, computed tomography (CT) images in the supine position had been acquired for treatment planning purposes; the images extended from the patient’s mid-thigh to beyond the top of the head (71.5 cm), with a reconstructed slice thickness of 2.5 mm (pixel size 0.98 mm × 0.98 mm). Target and avoidance structures were contoured as well as organs and tissues that corresponded to possible SMN sites, which included the “whole body” (*i.e.*, that captured in the CT image set), bladder, breasts, colon, liver, lungs, ovaries, skin, stomach, thyroid, uterus, rectum, red bone marrow, and a “remainder” that was created as a Boolean subtraction of the union of all specific SMN-site organs and tissues from the whole body. This remainder volume included the central nervous system. SMN risks from intracranial boost fields targeting the tumor resection bed or posterior fossa were not considered in this study because boost fields are small and bear much less importance for SMN risk than the CSI fields [10]. This study was performed under protocols approved by MD Anderson’s and AUBMC’s institutional review boards.

#### 2.1.1. Proton CSI

The MD Anderson standard proton treatment technique for pediatric CSI is administered with the patient positioned in the supine position [21]. The RBE of therapeutic protons for deterministic effects throughout the treatment volume was taken as 1.1 (ICRU 2007). Therefore, the absorbed dose from proton beams that corresponded to *D_Rx_* was 21.3 Gy. A treatment couch model had been added to the CT images to account for attenuation through the couch. The proton CSI treatment plan was created using a commercial treatment planning system (TPS; Eclipse, version 8.9; Varian Medical Systems, Inc., Palo Alto, CA, USA) [23]. The treatment unit was MD Anderson’s passive scattering proton therapy gantry (Probeat, Hitachi America, Ltd., Brisbane, CA, USA) that was described previously, along with the CSI delivery technique [9,23,24,25]. The proton CSI plan comprised five treatment fields: lower, middle, and upper posterior anterior spinal fields (LPA, MPA, and UPA) and right and left posterior oblique cranial fields (RPO and LPO). The original plan included two longitudinal shifts of the position of the abutment of the fields, but these shifts were not taken into consideration in this research study. The field characteristics are listed in Table 1.

**Table 1:** Treatment field characteristics for the proton CSI, with Monitor Units listed for *D_Rx_*. “Air gap” refers to the distance between the distal edge of most downstream component of the treatment unit (range compensator) and the proximal surface of the patient along the central beam axis.

Proton CSI	LPA	MPA	UPA	RPO	LPO
Monitor Units	2493	2594	2577	1349	1323
Nominal injection beam energy (MeV)	180	160	160	200	200
Range in patient (cm H_2_O)	14.2	10.9	11.4	15.3	15.8
Nominal SOBP width (cm)	7	6	5	16	16
Gantry angle (°)	180	180	180	255	105
Couch rotation angle (°)	0	0	0	2	358
Air gap (cm)	23	25	26	38	39
Aperture block thickness (cm)	6	4	4	6	6
Pre-collimated field size (cm × cm)	25 × 25	25 × 25	25 × 25	25 × 25	25 × 25
Collimated field, major axis (cm)	22.7	13.4	11.9	21.1	20.7
Collimated field, minor axis (cm)	6.1	4.8	5.5	16.7	16.9

Abbreviations: LPA = lower posterior anterior; MPA = middle posterior anterior; UPA = upper posterior anterior; RPO = right posterior oblique; LPO = left posterior oblique; SOBP = spread-out Bragg peak.

The TPS was used to calculate the in-field dose throughout the virtual geometry, including the patient and the treatment couch, with a treatment planning calculation grid of 2.5 mm. Equivalent dose from secondary neutrons was calculated in each voxel using the Monte Carlo Proton Radiotherapy Treatment Planning (MCPRTP) system [9,23,26,27]. The MCPRTP system was modified prior to this study to include a lattice tally instead of a mesh tally to calculate dose within the patient [28], an upgrade of the dose engine to version 2.7c of the Monte Carlo N-Particle eXtended code [29], more-specific isotopic materials cards that call cross-section tables when available, the most up-to-date data libraries, an improved multiple Coulomb scattering algorithm [30,31], a reduced bin size for more precise proton stopping power, and removal of triton and ^3^He tracking. The system was validated by comparing its results to the results of previous studies and measured data. These changes resulted in more accurate dosimetric calculations, faster simulation times, and reduced variance. The treatment-plan Digital Imaging and Communications in Medicine-Radiation Therapy (DICOM-RT) objects [32]—RT Image, RT Plan, RT Dose, and RT Structure Set—were exported to separate files and imported into MCPRTP to determine the secondary neutron absorbed dose. The geometric model included field-specific components of the treatment unit, including the structural and shielding materials, range modulator wheel, scattering foil, range shifter, collimating aperture, and range compensator [25,26], and the extent of the patient geometry in the planning CT image set. The CT number in each voxel was converted to a mass density and a material composition in the geometric model using the machine-specific calibration curve of the TPS [9,27].

#### 2.1.2. Photon CSI

The RT Image and RT Structure Set files from MD Anderson containing the containing the CT images and contoured volumes were de-identified [33,34], sent to AUBMC via encrypted and password-protected data transfer, and imported into the AUBMC TPS (Panther, version 4.72, Prowess Inc., Concord, CA, USA). Three-dimensional radiation therapy treatment fields were planned according to the standard of care for pediatric photon CSI at AUBMC. The photon CSI plan comprised four 6-MV treatment fields: LPA and UPA spinal fields and right and left lateral cranial fields (RL and LL) to be delivered using AUBMC’s clinical linear accelerator and multi-leaf collimator (Artiste, Siemens Medical Solutions USA, Inc., Malvern, PA, USA). Cranial and upper spinal fields were matched by rotating the collimator used in the cranial fields, and no couch rotation was applied. Calculation points were placed on the central axis of each field: at the midline for the brain field and at the anterior cord depth for each spinal field. The fields were planned to deliver 23.4 Gy to calculation points while keeping the dose at any point in the cord or brain below 115% of the dose at the calculation points. The RBE of photons was taken as 1, so this prescription corresponded to a *D_Rx_* of 23.4 Gy-RBE in common with the proton plan. At AUBMC, photon CSI is performed typically with a patient in the prone position, with the chin rotated out of the path of the upper spinal field. Because the CT image set had been collected at MD Anderson with the patient in the supine position, adjustments were made in the photon plan to remove the treatment couch and the patient’s chin from the TPS calculations to resemble the scenario in which the CT image set had been collected with the patient in the prone position. Field characteristics are listed in Table 2.

### 2.2. In-Field Equivalent Dose in Organs and Tissues

Mean organ doses were determined from voxelized dose distributions calculated by each TPS. The absorbed dose in each voxel from primary fields, *D_v_*(pri), in Gy was calculated throughout the extent of the CT image set. The equivalent dose in each voxel from primary fields, *H_v_*(pri), in Sv was the product of the mean radiation weighting factor, $\bar{w_{R}}$ , used for deterministic and stochastic effects and *D_v_*(pri), or:

(1) $$H_{v}\text{(pri)}=\bar{w_{R}}\cdot D_{v}\text{(pri)}$$

$\bar{w_{R}}$ was taken as 1 for primary photon fields, following the recommendation in International Commission on Radiological Protection (ICRP) Publication 92 [35]. For primary proton fields, $\bar{w_{R}}$ was estimated as the mean quality factor [36,37] at any point within the fields, or approximately 1.1. *H_T_*(pri) was calculated as the mass-weighted average of the equivalent dose in all voxels from primary fields within a particular organ or tissue, *T*, using the following equation:

(2) $$H_{T}=\frac{\sum_{v\text{ in }T}H_{v}\rho_{v}}{\sum_{v\text{ in }T}\rho_{v}}$$

where *ρ_v_* was the mass density of the tissue within a voxel. Finally, because the captured CT image set did not extend from the thighs to the feet, the low-dose regions inferior to the CT image set were not included in the red bone marrow, skin, remainder, and whole body volumes. To account for the effect of the lessening of *H_T_*(pri) values by the weights of missing voxels in these regions, mean doses for these volumes were reduced by multiplying them by the ratio of the mass of the patient in the CT image set to the mass of the patient’s entire body recorded in her clinical chart, or 0.726. Effectively, this approximation assumed that the dose in tissues inferior to the CT image set was negligible compared to the dose within the CT image set.

**Table 2:** Treatment field characteristics for the photon CSI, with Monitor Units listed for *D_Rx_*.

Photon CSI	LPA	UPA	RL	LL
Monitor Units	3008	2785	1245	1268
Gantry angle (°)	180	180	270	90
Collimator angle (°)	0	0	11	349
X1 jaw (cm)	−4.4	−2.9	−9.3	−10.9
X2 jaw (cm)	3.7	2.6	10.9	9.3
Y1 jaw (cm)	−4.1	−9.0	−10.0	−10.0
Y2 jaw (cm)	10.0	19.0	10.5	10.5
Depth of calculation point (cm H_2_O)	6.7	5.1	7.6	8.1
Effective square (cm)	8.5	7.4	17.3	17.3
Source-to-skin distance (cm)	100	100	92.9	92.7

Abbreviations: LPA = lower posterior anterior; UPA = upper posterior anterior; RL = right lateral; LL = left lateral.

### 2.3. Stray Radiation Equivalent Dose in Organs and Tissues from Proton CSI

For proton CSI, absorbed dose from secondary neutrons in each voxel, *D_v_*(n), was calculated throughout the patient’s body—both in-field and out-of-field—using the MCPRTP system. The neutron component of the in-field absorbed dose was calculated separately because of the higher $\bar{w_{R}}$ for neutrons than for protons. In proton beam radiotherapy, neutrons are produced in both the patient (“internal” neutrons) and treatment unit (“external neutrons”). In the MCPRTP system, *D_v_*(n) was calculated for external neutrons, *D_v_*(n_ext_), and internal neutrons, *D_v_*(n_int_), in separate simulations for each treatment field, and the values were then summed for all fields. The values for $\bar{w_{R}}$ were taken from the literature based on Monte Carlo simulations of children that most closely approximated the size of the 13-year-old girl in this study. The $\bar{w_{R}}$ for external neutrons, $\bar{w_{R\text{,ext}}}$ , was estimated as the mid-range value from a previous study in which the $\bar{w_{R}}$ values were calculated by the MCPRTP system for passive-scattering proton CSI for a 10-year-old girl [10], so that $\bar{w_{R\text{,ext}}}$ = 9.2. The $\bar{w_{R}}$ for internal neutrons, $\bar{w_{R\text{,int}}}$ , was estimated as the average value from a previous study in which the $\bar{w_{R}}$ values were calculated by MCPRTP system for neutrons produced within a small, adult-male phantom for passive-scattering proton CSI [14], so that $\bar{w_{R\text{,int}}}$ = 9.0. Those previous studies defined $\bar{w_{R}}$ according to the recommendation found in ICRP Publication 92 [35]. Equation (1) was used to calculate *H_v_*(n_ext_) and *H_v_*(n_int_), Equation (2) was used to calculate *H_T_*(n_ext_) and *H_T_*(n_int_), and these values were reduced as described in Section 2.2 for organs and tissues that extended beyond the CT image set. Finally, for each treatment field, the total organ equivalent dose from secondary neutrons, *H_T_*(n), was the sum of the external and internal neutron contributions, or:

*H_T_*(n) = *H_T_*(n_ext_) + *H_T_*(n_int_) (3)

The equivalent dose results for neutrons calculated by MCPRTP were reported per source particle, *H_v_*/sp in (Sv·sp^−1^). Therefore, it was necessary to normalize the secondary neutron *H_T_* values to the therapeutic dose in the following manner. A beam normalization volume (BNV) was contoured for each field encompassing voxels in the central region of the proton CSI target volume of each field and receiving approximately uniform absorbed dose. Additional separate MCPRTP simulations were performed in which *D_v_*(pri)/sp (in Gy·sp^−1^) was determined and carried through the methods above to calculate *H_v_*(pri)/sp and *H*_BNV_(pri)/sp. Finally, *H_T_* was calculated as the product of *H_T_*/sp and the number of source particles required for *H*_BNV_(pri)/sp from MCPRTP to match the *H*_BNV_(pri) from the TPS, or:

(4) $$H_{T}=\frac{H_{T}}{\text{sp}}\times \text{source particles}=\frac{H_{T}}{\text{sp}}\times \frac{{\left\{H_{\text{BNV}}\left(\text{pri}\right)\right\}}_{\text{TPS}}}{{\left\{\frac{H_{\text{BNV}}\left(\text{pri}\right)}{\text{sp}}\right\}}_{\text{MCPRTP}}}$$

### 2.4. Equivalent Dose in Organs and Tissues for Photon CSI

Dose calculation algorithms of TPSs for photon radiotherapy are accurate in the in-field region but inaccurate in the out-of-field region [38,39,40,41]. To determine out-of-field equivalent dose in organs and tissues that are sensitive to radiation carcinogenesis, we used three distinct approaches and compared the results: (1) based on TPS calculations only; (2) a volume-weighted average based on measurements in an anthropomorphic phantom; and (3) based on a an analytical model relating out-of-field dose to distance from the CSI field edge.

#### 2.4.1. Approach 1: TPS Calculations

In the first approach, we calculated the mean equivalent dose in organs and tissues for in-field, partially in-field, and out-of-field organs based only on the TPS calculation. To maintain consistency between the methods of calculating mean *H_T_* values, we exported the dose distribution calculated by the AUBMC TPS to DICOM RT Dose files, imported them into the MD Anderson TPS, and used the MD Anderson TPS to calculate *H_T_*, as described in Section 2.2.

#### 2.4.2. Approach 2: Volume-Weighted Average

In the second approach, we applied the volume-weighted averaging technique developed by Howell *et al.* [42]. Dose volume histogram (DVH) data were exported from the TPS. For organs and tissues that were entirely within the treatment field, the DVH data alone was used to determine the equivalent dose in organs and tissues, as described in Section 2.2. For organs that were entirely outside of the treatment field, the mean organ equivalent dose values were determined from our previous study in which thermoluminescent dosimeter (TLD) measurements were made within an anthropomorphic phantom receiving photon CSI at AUBMC [41]. The mean organ equivalent dose in each out-of-field organ was calculated as an average of the equivalent doses of all TLD measurements within the organ. For organs and tissues that were partially in the treatment field, as described by Howell *et al.*, a sensitivity analysis was performed to determine if the DVH data were sufficiently accurate for calculating the mean organ dose. Specifically, if adjusting *D_v_* in the voxels outside the 5% isodose surface by ±60% resulted in a greater than ±5% difference in *H_T_*, then *H_T_* for that organ was estimated based on a volume-weighted average that combined the TPS estimate of the in-field component of dose with a measured out-of-field component of dose. If not, *H_T_* for that organ was determined based on the *D_v_* values from the TPS.

#### 2.4.3. Approach 3: Model-Based Estimate

In the third approach, we applied an analytical model developed in our previous study [41] of absorbed dose *vs.* distance from the CSI field edge (*i.e.*, 50% isodose surface). Because the treatment unit for this study was the same as that in the previous study, the model and its fitted parameters were applied directly. For each voxel that was more than 1 cm outside of the field edge, the *D_v_* values were replaced by those calculated by the following equation:

(5) $$H_{v}=\bar{w_{R}}\cdot D_{Rx}\cdot {\left\{\frac{D_{v}}{D_{Rx}}\right\}}_{v,\text{model}}=\bar{w_{R}}\cdot D_{Rx}\cdot \frac{1\text{ Gy}}{100\text{ cGy}}\cdot \left[\frac{\alpha_{1}}{\sqrt{2\pi \sigma_{1}^{2}}}e^{-\frac{{\left(r-\mu_{1}\right)}^{2}}{2\sigma_{1}^{2}}}+\frac{\alpha_{2}}{\sqrt{2\pi \sigma_{2}^{2}}}e^{-\frac{{\left(r-\mu_{2}\right)}^{2}}{2\sigma_{2}^{2}}}\right]$$

where α_1_ = 224.8 cm-cGy/Gy, μ_1_ = −2.16 cm, σ_1_ = 1.57 cm, α_2_ = 224.3 cm-cGy/Gy, μ_2_ = −7.43 cm, and σ_2_ = 10.28 cm from the previous study.

To replace the TPS-calculated values with those calculated by the model, we used the following computational methods. The 50% isodose surface was converted to a contoured structure using the TPS. Then the DICOM-RT objects for the dose distributions and contoured structures were exported from the TPS to separate RT Dose and RT Structure Set formatted files, respectively. The coordinates and doses of each voxel in the RT Dose file and the coordinates of each vertex of the 50% isodose surface in the RT Structure Set file were read into an in-house code and processed using commercial software (MATLAB R2008b, The MathWorks, Inc., Natick, MA, USA). For each voxel that was both outside the 50% isodose surface and at a distance greater than 1 cm from the nearest vertex of the 50% isodose surface, the code replaced *H_v_* with the model-based estimate from Equation (5). The resulting position and dose data were converted back into a DICOM RT Dose file, and *H_T_* values were calculated as described in Section 2.2.

### 2.5. Risks of SMN Incidence and Mortality

The excess absolute lifetime risks of radiogenic cancer incidence, *I_T_*, and mortality, *M_T_*, were estimated for specific cancer sites that correspond to *T* using linear-no-threshold models:

(6) $$I_{T}=\left(\frac{I_{T}}{H_{T}}\right)\cdot H_{T}\text{ and }M_{T}=\left(\frac{M_{T}}{H_{T}}\right)\cdot H_{T}$$

in which *I_T_*/*H_T_* and *M_T_*/*H_T_* were risk coefficients derived from the age-, sex-, and organ-specific risk models of the BEIR VII Report of the National Research Council of The National Academies [43]. *I_T_*/*H_T_* and *M_T_*/*H_T_* coefficients for each *T* were interpolated linearly between values for a 10-year-old girl and a 15-year-old girl from Tables 12D-1 and 12D-2 in the BEIR VII Report. *H*_remainder_ was used to estimate the risk of other solid cancers, and *H*_red bone marrow_ was used to estimate the risk of leukemia. This risk model was used for all cancers with the following exceptions in which recommended risk coefficients were not provided in the BEIR VII Report. For non-melanoma skin cancer (NMSC), we applied values of *I*_skin_/*H*_skin_ and *M*_skin_/*H*_skin_ that we derived from ICRP Publication 60 [36], adjusting for age and sex, as described previously [10]. For *M*_thyroid_/*H*_thyroid_, we multiplied the interpolated value for *I*_thyroid_/*H*_thyroid_ by the proposed lethality fraction for thyroid cancer of 0.1 from ICRP Publication 60. Thus, we took into account all known radiogenic cancer risks.

The risk coefficients for all cancers except leukemia had been reduced by a dose and dose rate effectiveness factor (DDREF) of 1.5 that corrected the risk models for low dose (<100 mGy) or low dose rate (<0.01 mGy/min) exposures. Because the organ doses in this study were moderate to high, we removed the DDREF by increasing *I_T_*/*H_T_* and *M_T_*/*H_T_* by a factor of 1.5 for all cancers except leukemia. Similarly, we excluded DDREF for *I*_skin_/*H*_skin_ and *M*_skin_/*H*_skin_. The final values of *I_T_*/*H_T_* and *M_T_*/*H_T_*, after adjustment for DDREF, for a 13-year-old girl are listed in Table 3. 

**Table 3:** Risk coefficients for radiogenic cancer incidence, *I_T_*/*H_T_*, and mortality, *M_T_*/*H_T_*, for a 13-year-old girl, listed in terms of absolute risk (in percent) per equivalent dose (in Sv).

Cancer Site	*I_T_*/*H_T_* (%/Sv)	*M_T_*/*H_T_* (%/Sv)
Stomach	0.98	0.55
Colon	2.15	1.00
Liver	0.26	0.23
Lung	6.78	5.96
Breast	9.25	2.17
Uterus	0.49	0.11
Ovary	0.98	0.54
Bladder	2.07	0.58
Thyroid	3.25	0.33
Leukemia	0.80	0.52
NMSC	17.77	0.04
Other solid	6.82	2.93

Risks are reported in percentages, meaning the number of SMNs or SMN fatalities per 100 13-year-old girls treated with these methods.

## 3. Results

### 3.1. Absorbed Dose, RBE-Weighted Absorbed Dose, and Equivalent Dose

**Figure 1:**
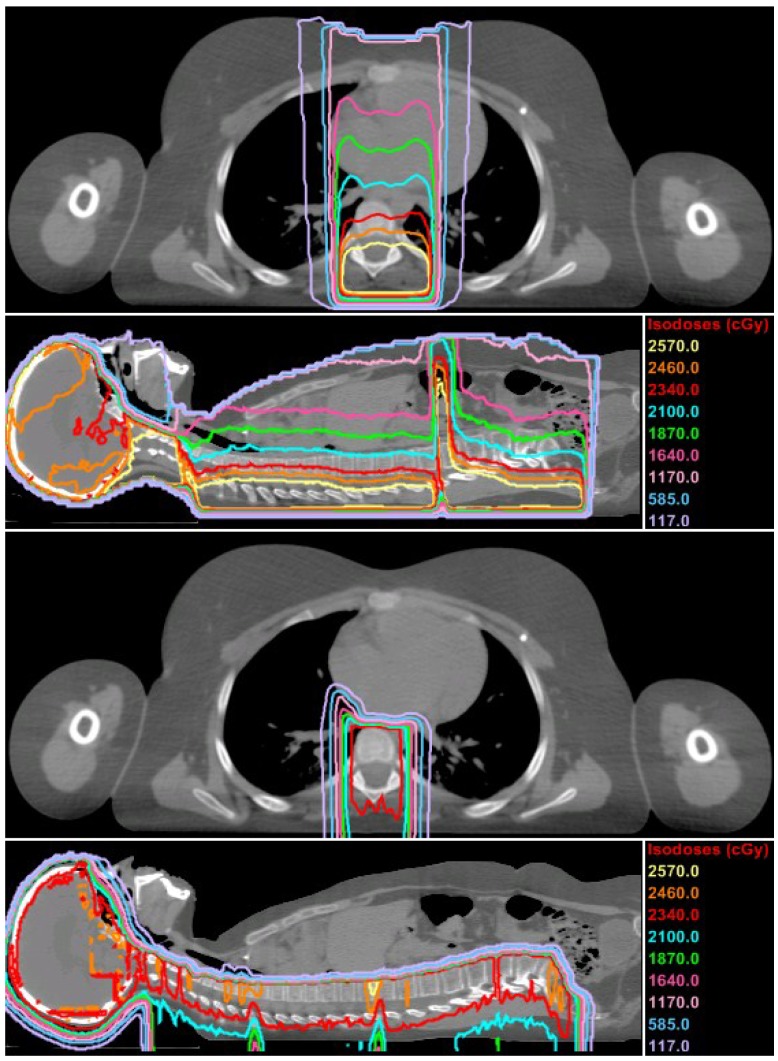
Predicted dose distributions from the primary fields in cGy-RBE for photon CSI at AUBMC (top half) and proton CSI at MD Anderson (bottom half) in axial and sagittal planes. The contour marked in red is the 100% isodose line, representing 23.4 Gy-RBE.

Figure 1 shows the distributions of *D_v_* of the therapeutic fields as calculated by the TPSs. In the axial plane shown, the proton CSI plan had a narrower (left to right) but deeper 100% isodose line than the photon CSI plan. These effects resulted from differences in standards of care at the two clinics, with AUBMC having wider lateral margins for photon CSI and MD Anderson including the distal portion of the vertebral body in the proton CSI target volume, both of which are to avoid a steep dose gradient in the vertebrae for patients that are still maturing and subsequently to reduce the risk of asymmetric bone growth [24].

Values of *H_T_* for proton CSI at MD Anderson are listed in Table 4, including dose from primary (therapeutic) fields and secondary neutrons. Primary proton fields were the main contributors to *H_T_* in the red bone marrow, remainder, whole body, skin, and lungs. These were also the organs and tissues that received the largest *H_T_*. The liver, thyroid, and colon received comparable contributions from proton fields and secondary neutrons, and secondary neutrons predominated *H_T_* in the stomach, breasts, uterus, bladder, and ovaries. In each organ and tissue, both external and internal neutrons were contributors to *H_T_*, and *H_T_* from external neutrons was between 1.4 and 4.4 times that from internal neutrons. Apart from the whole body volume, the organs and tissues receiving the highest *H_T_* in proton CSI were the red bone marrow, remainder, skin, and lungs.

**Table 4:** Mean organ equivalent dose, *H_T_*, for proton CSI of a 13-year-old girl at MD Anderson. The values in the table include *H_T_* attributable to therapeutic protons, neutrons generated in the treatment unit (“external neutrons”), and neutrons generated in the patient (“internal neutrons”). The TPS does not estimate statistical uncertainty in dose, and the statistical uncertainties for neutron *H_T_* values were less than 3%.

*H_T_* (Sv)				
Organ or Tissue	Primary Protons	External Neutrons	Internal Neutrons	Total
Stomach	0.01	0.34	0.14	0.49
Colon	0.08	0.27	0.11	0.46
Liver	0.39	0.38	0.12	0.89
Lungs	1.82	0.48	0.17	2.47
Breasts	0.00	0.32	0.07	0.39
Uterus	0.00	0.21	0.08	0.29
Ovaries	0.00	0.18	0.07	0.25
Bladder	0.00	0.13	0.06	0.19
Thyroid	0.45	0.44	0.31	1.20
Red bone marrow	4.41	0.24	0.14	4.79
Skin	2.40	0.27	0.12	2.79
Remainder	2.84	0.27	0.16	3.27
Whole body	2.52	0.27	0.15	2.94

Values of *H_T_* for photon CSI at AUBMC are listed in Table 5 for each of the three methods of estimating out-of-field dose. In photon CSI, the uterus, ovaries, and bladder were entirely out-of-field, and all other volumes were partially in-field. The sensitivity analysis (see Section 2.4.2) for each of the partially in-field organs revealed that the TPS was sufficiently accurate for calculating *H_T_* values. This was true even for the breast, in which only 12% of the volume was within the 5% isodose surface. Therefore, in the volume-weighted average approach, *H_T_* was modified only for the uterus, ovaries, and bladder, as shown in Table 5. The model-based approach for replacing the out-of-field dose resulted in higher *H_T_* values. Those increases in *H_T_* were slight in the whole body, stomach, remainder, red bone marrow, and skin (percent difference ≤10%); moderate in the colon, liver, and lungs (10% < percent difference ≤ 50%), and large in the bladder, uterus, breasts, and ovaries (percent difference >50%). Compared to the model-based approach, the TPS calculation underestimated *H*_breasts_ by more than a factor of 2. The volume-weighted approach resulted in higher *H*_uterus_, *H*_ovaries_, and *H*_bladder_ values that were higher than those of the TPS calculations but lower than those of the model-based approach. Because it was entirely in-field, the choice of methods had no effect on the reported thyroid dose. The organs and tissues receiving the highest *H_T_* in photon CSI at AUBMC were the thyroid, stomach, colon, red bone marrow, and lungs.

After accounting for out-of-field dose, *H_T_* values were higher for photon CSI at AUBMC than for proton CSI at MD Anderson in all organs and tissues except the skin, which had approximately equal *H_T_*. The red bone marrow, remainder, and whole body organs also had *H_T_* values for photon CSI that were within a factor of 2 of those of proton CSI. Other radiosensitive organs and tissues had *H_T_* values for photon CSI that were factors of 2.3 to 20.1 higher than those of proton CSI.

**Table 5:** Mean organ equivalent dose, *H_T_*, for photon CSI of a 13-year-old girl at AUBMC. Three distinct methods were compared (see Section 2.4). The values from the far right column were used in SMN risk assessments and comparisons with proton CSI at MD Anderson.

*H_T_* (Sv)			
Organ or Tissue	TPS Only	Volume-Weighted Averaging Technique	TPS in-Field & Model Out-of-Field
Stomach	9.48	9.48	9.73
Colon	6.12	6.12	6.82
Liver	4.64	4.64	5.33
Lungs	4.78	4.78	5.59
Breasts	0.72	0.72	1.57
Uterus	0.02	0.60	1.11
Ovaries	0.02	0.60	1.70
Bladder	0.03	0.60	1.10
Thyroid	16.75	16.75	16.75
Red bone marrow	5.49	5.49	5.88
Skin	2.58	2.58	2.80
Remainder	4.77	4.77	4.91
Whole body	4.53	4.53	4.64

### 3.2. Risk of SMN Incidence and Mortality

**Figure 2:**
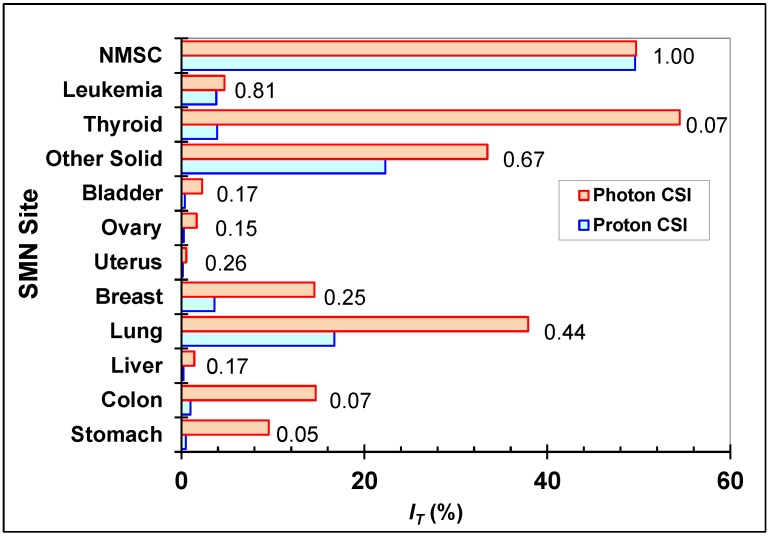
Site-specific predicted lifetime attributable risks of SMN incidence after photon CSI at AUBMC or proton CSI at MD Anderson. Other solid cancer risks were based on *H*_remainder_, and leukemia risks were based on *H*_red bone marrow_. These risk estimates took into account both primary and stray radiation. The ratios of the risks (proton CSI:photon CSI) are shown for each SMN site.

*I_T_* values from photon CSI at AUBMC and proton CSI at MD Anderson are shown in Figure 2, as are the ratios of *I_T_* for proton CSI to *I_T_* for photon CSI. The SMN with the highest *I_T_* values for photon CSI were thyroid cancer, NMSC, lung cancer, and other solid cancers. Those with highest *I_T_* values for proton CSI were NMSC, other solid cancers, and lung cancer. Differences in *I_T_* of 5% or more between the two treatments were found for thyroid, lung, colon, breast, stomach, and other solid cancers; and differences in *I_T_* for the other cancers were less than 2%. Ratios of *I_T_* were less than 1 for all cancers except NMSC and were smallest for stomach, colon, and thyroid cancers. The ratio of the sums of *I_T_* for all cancers was 0.46 when including NMSC and 0.30 when excluding NMSC. NMSC accounted for one-quarter and one-half of the sums of *I_T_* in photon CSI and proton CSI, respectively. In photon CSI, only one-tenth of the total risk of SMN incidence was from out-of-field dose, but in proton CSI, one-quarter of the total risk of SMN incidence was from stray radiation.

**Figure 3:**
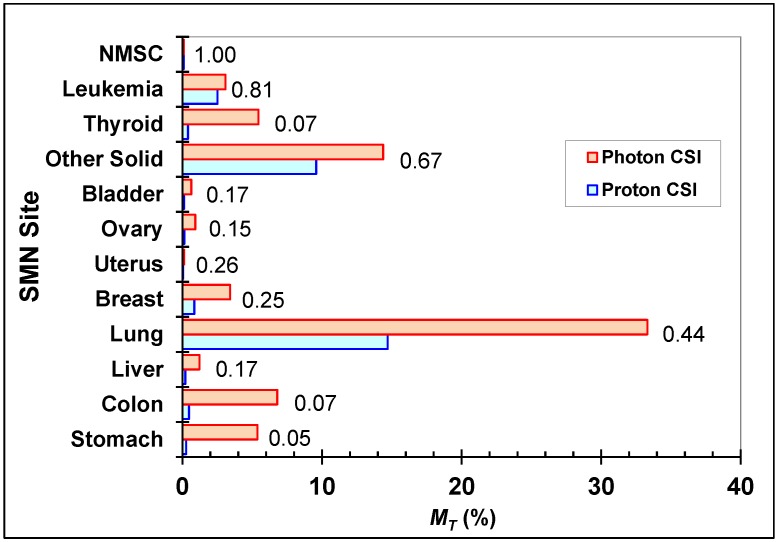
Site-specific predicted lifetime attributable risks of SMN mortality after photon CSI at AUBMC or proton CSI at MD Anderson. Other solid cancer risks were based on *H*_remainder_, and leukemia risks were based on *H*_red bone marrow_. These risk estimates took into account both primary and stray radiation. The ratios of the risks (proton CSI:photon CSI) are shown for each SMN site.

*M_T_* values from photon CSI at AUBMC or proton CSI at MD Anderson are shown in Figure 3 along with the ratios of *M_T_* for proton CSI to *M_T_* for photon CSI. The SMN sites of greatest concern for a radiation-related fatality were lung cancer and other solid cancers in both cases. The ratios of risk for each SMN site were identical to those for incidence. Differences in *M_T_* of 5% or more were found for lung, colon, stomach, thyroid, and other solid cancers. The ratio of risk of SMN mortality for all cancer sites was 0.39, and the risk of fatality from NMSC was negligible. In photon CSI, one-eighth of the total risk of SMN mortality was from out-of-field dose, and in proton CSI, one-quarter of the total risk of SMN mortality was attributable to stray radiation.

## 4. Discussion

We performed an inter-institution comparison study on the predicted lifetime attributable risks of SMN incidence and mortality for a 13-year-old girl receiving photon CSI at an academic cancer center in the Middle East with those for the same patient receiving proton CSI at an academic cancer center in the U.S., taking into account both primary and stray radiation and all known radiogenic cancers. We found that the risks of incidence or mortality for proton CSI were approximately half those for photon CSI. With proton CSI, the absolute risks of SMN incidence were especially reduced for thyroid, lung, colon, breast, stomach, and other solid cancers compared to photon CSI. The specific SMN of greatest concern for a radiation-related fatality was lung cancer, accounting for half the risk in each case. It was also lung cancer for which proton therapy yielded the greatest reduction in absolute SMN mortality risk. Stray radiation contributed to the incidence and mortality risks in both treatment modalities and was especially important in proton CSI, even though the total risk remained lower in all cancer sites. This study demonstrated the feasibility of comparative inter-institutional whole-body dose and risk assessment capabilities.

The predicted lifetime absolute attributable risks of SMN incidence and mortality in this study were high. This was true even for SMNs that originate outside the craniospinal axis. In a previous study for a 4-year-old boy undergoing proton CSI or photon CSI at MD Anderson [19], we found that high predicted risk of SMN incidence for the first 30 years after exposure were comparable to but lower than the observed excess risk of SMN incidence in the exposed population of a large-scale epidemiological study (Childhood Cancer Survivor Study) [44]. Like us, Meadows *et al.* found that the probability of NMSC incidence was high. As time after exposure increases, the treatment-related incidence and mortality rates for their cohort likely will increase. In a study involving 10 pediatric patients, Brodin *et al.* [15] also predicted high excess absolute risks of SMN incidence (>40% over a lifetime) following photon CSI and moderate excess risks of this outcome (<20% over a lifetime) following intensity-modulated proton CSI. Therefore, the findings of Brodin *et al.* agree with our high predicted risk of SMN incidence our patient and decreased risk of SMN incidence by using proton instead of photon CSI. In a previous study for a cohort of 17 patients receiving proton CSI or photon CSI within MD Anderson, we found the ratios of SMN risks from proton CSI *versus* photon CSI to range between 0.10 to 0.22 for incidence and between 0.20 and 0.53 for mortality [16]. Our ratio for mortality in this study (0.39) fell within the range of values of the previous study, but our ratio for incidence (0.46) did not. This difference is likely due to our more comprehensive estimates of radiation carcinogenesis in this study that included NMSC, leukemia, and other solid cancers. A direct comparison between SMN incidence and mortality due to photon CSI at MD Anderson and photon CSI at AUBMC is not easily obtained because of the differing methods between the two studies.

For the patient and treatment tested here, one-sixth of the risks of SMN incidence and mortality in proton CSI were due to external neutrons. This finding confirms a similar previous result for a 3-year-old boy [14]. By neglecting the equivalent dose from external neutrons, we can obtain a first approximation for the decline in risks that may result from using to intensity-modulated proton therapy (IMPT). IMPT is being used increasingly for pediatric radiotherapy [45]. Some existing and most new proton therapy installations under construction are designed to deliver magnetically scanned proton beams capable of IMPT. With these units, patients receive negligible external neutron exposures compared to therapeutic proton and internal neutron exposures. In previous study for a 3-year-old boy [14], we expanded the methods of study into the reduction of lifetime SMN risk that may be achievable by proton therapy by Miralbell *et al.* [17] to include contributions from secondary neutrons. In that study, passively-scattered proton CSI reduced the risk of SMN incidence compared to conventional photon CSI by a factor of 10.7, and scanned-beam proton CSI (a closer approximation to IMPT) was estimated to increase this factor to 12.4. The possibility of a further reduction in SMN risk with the use of IMPT should be explored more systematically.

We estimated the predicted risks of incidence of NMSC and found them to be approximately equal between proton and photon CSI. NMSC is the most common treatment-related subsequent neoplasm in childhood cancer survivors [44] and has been found in survivors as young as 7 years and as old as 46 years, with 10% of the tumors appearing outside the treatment field among patients receiving radiotherapy [46]. Of particular concern was a finding by Armstrong *et al.* [47] that NMSC is often followed by other SMN. Among childhood cancer survivors who had received radiotherapy within the previous 15 years and who had two or more SMN, the risk of multiple subsequent neoplasms was twice as high for those whose first subsequent neoplasm was an NMSC than for those whose first subsequent neoplasm was not. For these reasons, although NMSC is rarely fatal, it was important to estimate NMSC risk for this study.

Children are particularly susceptible to SMN and some other late effects because of the radiation sensitivity of their organs and tissues, the proximity of non-target organs and tissues to the treatment fields, and the ongoing development of their organs [48,49,50]. Therefore, understanding the role of advanced techniques to reduce radiotherapy-induced SMN risk is especially important for children. Furthermore, childhood cancer survivors generally have a high likelihood of long-term survival. In our study, we predicted that SMNs are avoidable by applying proton CSI rather than photon CSI. If advanced techniques such as proton therapy are proven to be superior for reducing late effects in children of all ages, countries in which proton therapy is not available may be inspired to devise creative means of making these modalities available to their children, for example, by sending their pediatric patients abroad for proton therapy [51]. However, then they must overcome a difficult obstacle, namely, that arranging for proton therapy in another country can take weeks or even months, which is inconsistent with the need to start irradiation within 30 days of surgery. Furthermore, we acknowledge that some countries lack resources for oncology care in general, and providing proton CSI for children may be low on their priority lists. Still, as the number of proton therapy clinics is growing rapidly, sending children to a centralized proton radiotherapy clinic is becoming more plausible.

The main limitations of our study are associated with the risk models. The uncertainties in risk are large and difficult to estimate, owing in part to the imprecise understanding of the applicability of DDREF in fractionated radiotherapy. The risk model has other inherent limitations. The models were derived from data from a general population, mainly atomic bomb survivors, and the application of these risk models to cancer patients receiving therapeutic doses introduces uncertainty in the risk predictions that is difficult to quantify [14,43,52,53]. Furthermore, the comprehensive SMN risk models used in this study were intended to be applied to a population, not to individuals, and the mortality risk coefficients were meant for health care in the United States. We acknowledge these systematic uncertainties and note that they limit our ability to predict risk with accuracy. However, our ratio-of-risk results and our general findings are less affected by these uncertainties than our absolute risk values.

## 5. Conclusions

In summary, this case study demonstrated the feasibility of inter-institutional whole-body dose and risk assessments and provided further evidence that proton radiotherapy reduces the risk of late effects for children with brain cancer *vs.* photon therapy. Specifically, when considering all radiogenic cancers, we found that a 13-year-old girl receiving proton CSI incurred much lower predicted risks of SMN incidence and mortality than she would if receiving photon CSI. The SMN of greatest concern for mortality was lung cancer. The results of our study suggest that the risk of radiogenic cancer may be reduced and quality of life improved for children needing CSI in countries in which proton therapy is not available if proton therapy is accessible to them; however, more research is needed to confirm these findings in patients of various ages and both sexes.
